# Supplementary material for: At-Home High-Intensity Interval Training for Individuals with Paraplegia Following Spinal Cord Injury: A Case Series
Source: J Phys Med Rehabil. Author manuscript; Available in PMC 2026 Mar 10. (PMC12970954; doi:10.33696/rehabilitation.7.056)
Supplement: JPMR-25-056-Supplementary_Files [file NIHMS2149763-supplement-JPMR-25-056-Supplementary_Files.zip › Supplementary_Table 1.docx]

# SCI Exercise Self-Efficacy Scale (ESES)

Please tell us how confident you are with regard to carrying out regular physical activities.

# I am confident…

1. …that I could always overcome barriers and challenges with regard to exercise if I try hard enough.

| □  Not at all true | □  Hardly True | □  Moderately True | □  Exactly True |
| --- | --- | --- | --- |

1. …that I could find the means and ways to exercise and be physically active.

| □  Not at all true | □  Hardly True | □  Moderately True | □  Exactly True |
| --- | --- | --- | --- |

1. …that it is easy for me to accomplish my activity and exercise goals.

| □  Not at all true | □  Hardly True | □  Moderately True | □  Exactly True |
| --- | --- | --- | --- |

1. …that when I am confronted with a barrier to exercise I could usually find several solutions to overcome this barrier.

| □  Not at all true | □  Hardly True | □  Moderately True | □  Exactly True |
| --- | --- | --- | --- |

1. …I could exercise even when I am tired.

| □  Not at all true | □  Hardly True | □  Moderately True | □  Exactly True |
| --- | --- | --- | --- |

1. I could exercise even when I am feeling depressed.

| □  Not at all true | □  Hardly True | □  Moderately True | □  Exactly True |
| --- | --- | --- | --- |

1. …that I could exercise even without the support of my family or friends.

| □  Not at all true | □  Hardly True | □  Moderately True | □  Exactly True |
| --- | --- | --- | --- |

1. …that I could exercise without the help of an exercise therapist.

| □  Not at all true | □  Hardly True | □  Moderately True | □  Exactly True |
| --- | --- | --- | --- |

1. …that I could be physically active despite my spinal cord injury

| □  Not at all true | □  Hardly True | □  Moderately True | □  Exactly True |
| --- | --- | --- | --- |

1. …that I could exercise even if I had no access to a gym or training facility.

| □  Not at all true | □  Hardly True | □  Moderately True | □  Exactly True |
| --- | --- | --- | --- |

# Reference:

Kroll, T., Kehn, M., Ho, P-S., Groah, S (2007). The SCI Exercise Self Efficacy Scale (ESES): Development and Psychometric Properties. *International Journal of* Behavioral Nutrition and Physical Activity; 4:34
